# Beneficial cardiovascular effects of reducing exposure to particulate air pollution with a simple facemask

**DOI:** 10.1186/1743-8977-6-8

**Published:** 2009-03-13

**Authors:** Jeremy P Langrish, Nicholas L Mills, Julian KK Chan, Daan LAC Leseman, Robert J Aitken, Paul HB Fokkens, Flemming R Cassee, Jing Li, Ken Donaldson, David E Newby, Lixin Jiang

**Affiliations:** 1 Centre for Cardiovascular Sciences, Edinburgh University, Edinburgh, UK; 2 Centre for Environmental Health, National Institute for Public Health and the Environment (RIVM), Bilthoven, the Netherlands; 3 Institute of Occupational Medicine, Edinburgh, UK; 4 Fuwai Hospital, Chinese Academy of Medical Sciences & Peking Union Medical College, Beijing, PR China

## Abstract

**Background:**

Exposure to air pollution is an important risk factor for cardiovascular morbidity and mortality, and is associated with increased blood pressure, reduced heart rate variability, endothelial dysfunction and myocardial ischaemia. Our objectives were to assess the cardiovascular effects of reducing air pollution exposure by wearing a facemask.

**Methods:**

In an open-label cross-over randomised controlled trial, 15 healthy volunteers (median age 28 years) walked on a predefined city centre route in Beijing in the presence and absence of a highly efficient facemask. Personal exposure to ambient air pollution and exercise was assessed continuously using portable real-time monitors and global positional system tracking respectively. Cardiovascular effects were assessed by continuous 12-lead electrocardiographic and ambulatory blood pressure monitoring.

**Results:**

Ambient exposure (PM_2.5 _86 ± 61 *vs *140 ± 113 μg/m^3^; particle number 2.4 ± 0.4 *vs *2.3 ± 0.4 × 10^4 ^particles/cm^3^), temperature (29 ± 1 *vs *28 ± 3°C) and relative humidity (63 ± 10 *vs *64 ± 19%) were similar (P > 0.05 for all) on both study days. During the 2-hour city walk, systolic blood pressure was lower (114 ± 10 *vs *121 ± 11 mmHg, P < 0.01) when subjects wore a facemask, although heart rate was similar (91 ± 11 *vs *88 ± 11/min; P > 0.05). Over the 24-hour period heart rate variability increased (SDNN 65.6 ± 11.5 *vs *61.2 ± 11.4 ms, P < 0.05; LF-power 919 ± 352 *vs *816 ± 340 ms^2^, P < 0.05) when subjects wore the facemask.

**Conclusion:**

Wearing a facemask appears to abrogate the adverse effects of air pollution on blood pressure and heart rate variability. This simple intervention has the potential to protect susceptible individuals and prevent cardiovascular events in cities with high concentrations of ambient air pollution.

## Introduction

Air pollution, and especially traffic-derived particulate matter [1], is now established as a major cause of cardiorespiratory morbidity and mortality [2-4]. Epidemiological studies have shown that chronic air pollution exposure is associated with the degree of atherosclerosis [5,6], and the risk of cardiovascular events [7]. Acute exposure causes exacerbation of existing cardiorespiratory conditions leading to an increase in hospital admissions [8] and deaths [9].

The mechanisms of these associations are unclear but recent controlled exposure studies have demonstrated that air pollution causes vascular endothelial dysfunction [10], arterial vasoconstriction [11], increased blood pressure [12] and myocardial ischaemia [13]. Observational studies have also suggested that air pollution exposure impairs regulation of the autonomic nervous system and reduces heart rate variability [14,15]. A combination of these effects is likely to account for the increase in cardiovascular events seen following exposure to air pollution. There is therefore a need to consider approaches that can reduce ambient air pollution exposure on both a personal and societal level.

In Beijing China, particulate matter (particle diameter < 10 μm; PM_10_) air pollution averages around 150 μg/m^3 ^and, in 2006, levels exceeded the World Health Organisation recommended national standards (PM_10_concentration < 50 μg/m^3^) on 241 out of 365 days [16]. Despite considerable efforts to improve air quality, pollution remains the single largest environmental and public health issue affecting Beijing. The extensive use of coal and the growing number of motor vehicles (estimated 3.3 million vehicles on the roads in August 2008) have contributed to air pollution. In addition, the city's geographical location exacerbates the problem with the surrounding mountain ranges impeding air circulation and dispersion of pollutants.

Increasing concern relating to the health effects of air pollution has led many individuals to use facemasks to reduce personal exposure. The efficiency of these masks and the potential cardiovascular benefits on people exposed to urban air pollution has yet to be established. The aims of this study were to assess the efficacy of facemasks in removing potentially hazardous particulate air pollution and to determine the potential cardiovascular benefits of a simple facemask in a polluted urban environment.

## Methods

### Subjects

Fifteen healthy volunteers were recruited from the Fuwai Hospital, Beijing in August 2008. All subjects were non-smokers, received no regular medication, and had no intercurrent illnesses. All subjects gave their written consent to participate in the study, which was reviewed and approved by the local ethics committee, in accordance with the Declaration of Helsinki.

### Assessment of mask efficacy

Masks designed for use by cyclists, pedestrians and occupational settings were tested for penetrance of fresh diesel exhaust particulate. Diesel engine exhaust was generated from the idling (1500 rpm) engine (F3M2011, Deutz Ag, Köln, Germany) of a 35 KVA generator (Bredenoord, Apeldoorn, Netherlands). The exhaust was diluted with filtered air to obtain a mass concentration of 75 ± 12 μg/m^3 ^(as measured by gravimetric analysis) and a particle number concentration of 500,000 particles/cm^3 ^(condensation particle counter [CPC] model 3022, TSI Instruments, High Wycombe, UK). Sections of each mask filter were mounted in a filter holder. After 5 min of baseline measurements, filters were introduced between the exhaust and the CPC that sampled at a flow rate of 1.5 L/min. Particle number was recorded for 5 min and penetrance defined as the percentage of particles passing through the filter compared to baseline.

### Study design

**Figure 1:**
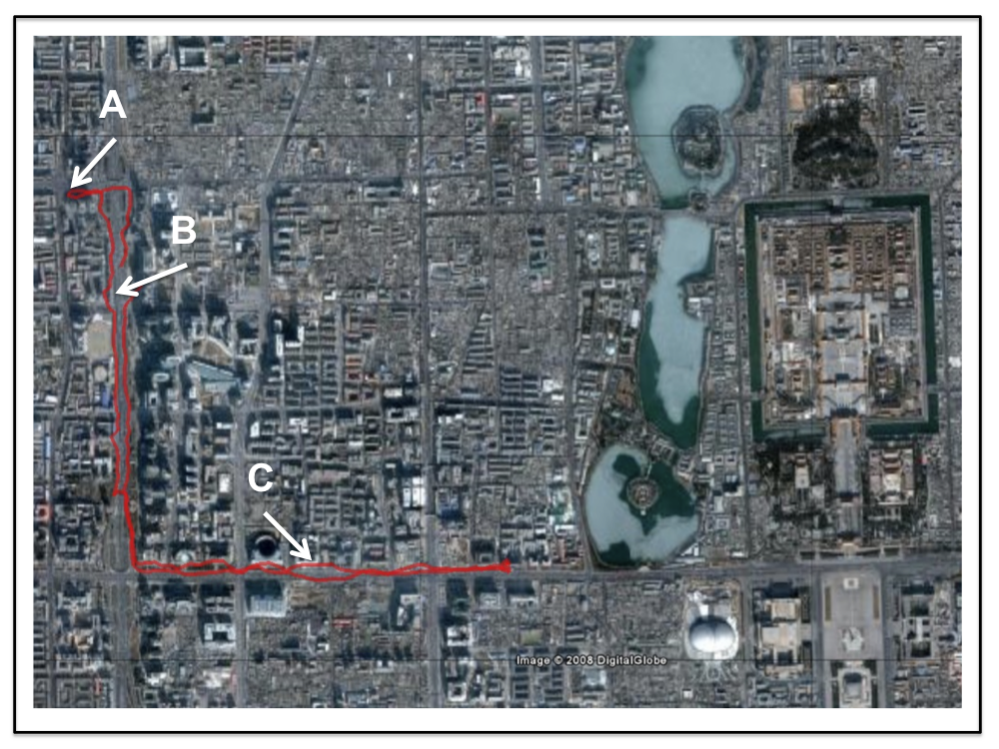
**City centre route chosen in central Beijing**. A representative recording from the GPS device contained in the monitoring backpack is shown. The walk goes from the Fuwai Hospital (A), along the inner ring road (B) and towards the city centre (C) before turning back. Image courtesy of Google™ Earth.

All 15 subjects attended the Fuwai Hospital on two occasions, each at least one week apart, during August 2008. In a randomised open-label controlled cross-over study, subjects were randomised to wear no mask or a highly efficient facemask filter (Dust Respirator 8812, 3 M, St Paul USA). When randomised to wear the facemask, subjects were asked to wear the mask for 24 hours prior to the study day and 24 hours of the study day. Subjects were asked to wear the mask at all times when outside, and as much as possible whilst indoors. On the study day, subjects were asked to walk for 2 hours in a city centre location (Figure 1) along the inner ring road in Beijing between 8 and 10 am.

### Pollution and activity monitoring

Personal exposure to air pollutants was monitored using a collection of portable monitoring equipment mounted in a backpack. Particle mass concentration (particle diameter < 2.5 μm; PM_2.5_) was measured in using a light-scattering nephelometric method using a DataRAM monitor (pDR-1500, Thermo Scientific, Franklin, USA). Particle number was measured using a handheld condensation particle counter (CPC 3007, TSI Instruments Ltd, High Wycombe, UK). Ambient temperature and relative humidity were recorded using a sensor on the outside of the backpack (Omegaette^® ^HH-314, Omega Engineering Ltd, Connecticut, USA). Gaseous pollutants were measured using a multigas analyser (X-am 7000, Dräger Safety, Pittsburgh, USA) measuring carbon monoxide (CO), nitrogen dioxide (NO_2_) and sulphur dioxide (SO_2_) using electrochemical sensors with a sensitivity of 1 part per million.

Physical activity was assessed using a portable global positioning system (GPS) monitor secured to the outside of the bag (eTrex Summit HC, Garmin, USA). This recorded the route taken by volunteers, their total distance travelled, and average speed. This information was used, along with baseline anthropometric measurements, to calculate the energy expended during the walk in kilocalories and metabolic equivalents (METS).

### Holter monitoring

Subjects were fitted with a 12-lead continuous electrographic Holter monitor (Lifecard 12, Spacelabs, UK) at the beginning of the study day for 24 hours. Holter electrographic traces were analysed using DelMar Reynolds proprietary software packages by two blinded observers. The quality of the electrocardiographic trace was manually inspected before arrhythmias were automatically detected using the Pathfinder software package. Identified arrhythmias were then individually inspected, verified or deleted as appropriate. Average heart rate and heart rate variability in both time and frequency domains were analysed using the HRV Tools software package, with identified arrhythmias excluded from this analysis.

### Ambulatory blood pressure

Subjects were fitted with an ambulatory blood pressure monitor (Model 90217, Spacelabs, UK) at the beginning of the study day. Blood pressure was recorded at the left brachial artery every 15 minutes during the 2-hour walk, every 30 minutes for the rest of the daytime (07:00 to 22:00), and every hour overnight (22:00 – 07:00).

### Symptom questionnaire

Subjects were asked to complete a symptom questionnaire using a visual analogue scale at the beginning of the study day, after the 2-hour walk and at the 24-hour visit. They were asked to record any physical symptoms, as well as report a perception of the degree of pollution and the tolerability of the mask.

### Data analysis and statistical methods

Subjects were randomised to wearing a mask on their first or second visit using a random number generator. All data are expressed as mean (95% confidence interval [CI]) unless otherwise stated. The symptom questionnaire was based on a visual analogue scale. Scores were converted into a percentage, and analysed using 2-way analysis of variance (ANOVA) with repeated measures using time and the mask intervention as variables. The occurrences of arrhythmias during the 24-hour monitoring period were compared using the Wilcoxon matched pairs method. All other parameters were evaluated using paired Student's *t*-tests. Statistical significance was taken at the 5% level. All data were analysed using GraphPad Prism (Version 4 for Macintosh, GraphPad Software, San Diego, USA) on a Macintosh personal computer.

## Results

### Mask efficiency

**Figure 2:**
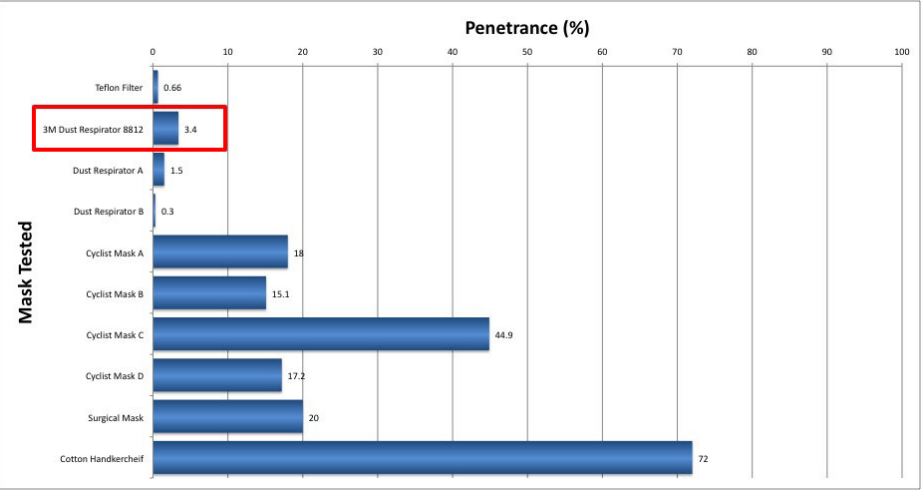
**Penetrance of commercially available filters: 3 M Dust Respirator 8812, Dust Respirators A and B, Cyclist Masks A to D**. The Teflon filter is an industry standard filter for aerosol studies included as a control. Cotton handkerchiefs and surgical masks are often seen worn in public areas in parts of Asia.

Mask penetrance was highly dependent on mask type (Figure 2). The 3 M Dust Respirator (Model 8812, 3 M, St Pauls, USA) was selected for the intervention study as it provided good filtration performance and was extremely efficient and comfortable to wear.

### Intervention study

Fifteen subjects (20–45 years) completed the study. Subjects were predominantly female (13:2) with a mean height of 164 cm (95% CI, 160 – 167), weight of 55 kg (95% CI, 50 – 60) and body mass index of 20.5 kg/m^2 ^(95% CI, 19.3 – 21.7). There were no differences (P > 0.05 for all parameters) in ambient pollution exposure during the 2-hour walk between the two visits (Table 1). Based on the measured penetrance of 3.4%, assuming a perfect facial fit and similar flow rates, we predict that the particle count to which subjects were exposed when wearing a mask was reduced to just 795 (95% CI, 726 – 864) particles/cm^3^.

**Table 1:** Exposure characteristics during city centre walks.

	Without Mask	With Mask
PM_2.5_, μg/m^3^	86 (52 – 120)	140 (77 – 203)
Particle count, number/cm^3^	24184 (22061 – 26306)	23379 (21350 – 25409)
CO, number of peaks	6.2 (3.2 – 9.3)	3.3 (1.3 – 5.2)
NO_2_, number of peaks	Nil	Nil
SO_2_, number of peaks	Nil	Nil
Temperature, °c	29.2 (28.6 – 29.8)	28.1 (26.3 – 29.9)
Relative humidity, %	63 (58 – 68)	64 (53 – 74)

Data expressed as mean (95% confidence interval).

P > 0.05 compared to control (without mask) day for all, Student's paired t-test.

There were no differences in 24-hour average heart rate or blood pressure during the two study days (Table 2). Holter analysis revealed an increased SDNN (65.6 ± 11.5 *vs *61.2 ± 11.4 ms, P < 0.05) and LF-power (919 ± 352 *vs *816 ± 340 ms^2^, P < 0.05) over the 24 hours when subjects wore the mask. There were no clinically relevant arrhythmias recorded in any subject (Table 3).

**Table 2:** 24-hour ambulatory blood pressure monitoring and Holter analysis for heart rate variability with each visit.

		Without Mask	With Mask
24 hour	SBP, mmHg	106 (100 – 112)	106 (101 – 111)
	DBP, mmHg	69 (66 – 73)	70 (67 – 73)
	MAP, mmHg	82 (79 – 86)	82 (78 – 86)
	Heart rate, bpm	74 (70 – 77)	72 (68 – 76)

Night	SBP, mmHg	100 (93 – 107)	101 (95 – 106)
	DBP, mmHg	63 (60 – 67)	64 (61 – 67)
	MAP, mmHg	76 (73 – 79)	75 (71 – 79)
	Heart rate, bpm	64 (61 – 67)	61 (58 – 65)

Day	SBP, mmHg	110 (104 – 116)	109 (104 – 114)
	DBP, mmHg	73 (69 – 76)	73 (70 – 76)
	MAP, mmHg	85 (81 – 88)	85 (81 – 89)
	Heart rate, bpm	79 (74 – 84)	78 (73 – 82)
Heart rate variability	Data validity, %	95.9	95.0
	Average NN interval, ms	829 (789 – 869)	850 (805 – 896)
	pNN50, %	15.9 (10.7 – 21.0)	17.9 (14.2 – 21.6)
	RMSSD, ms	35.1 (29.2 – 41.0)	37.1 (32.2 – 42.0)
	SDNN, ms	61.2 (54.9 – 67.5)	65.6* (59.0 – 72.2)
	Triangular Index	12.9 (11.9 – 13.9)	13.8 (13.0 – 14.5)
	LF-power, ms^2^	816 (628 – 1004)	919* (717 – 1122)
	HF-power, ms^2^	460 (325 – 595)	485 (400 – 569)
	LFn, ms	62.8 (56.7 – 68.9)	64.5 (60.6 – 68.4)
	HFn, ms	29.2 (25.5 – 32.8)	30.0 (27.0 – 33.1)
	HF/LF ratio	0.738 (0.507 – 0.970)	0.680 (0.519 – 0.842)

All data expressed as mean (95% confidence interval). *P < 0.05 compared to control (no mask) day, Student's paired t-test.

**Table 3:** Arrhythmia analysis from 24-hour Holter electrocardiograms.

	Without Mask	With Mask
Pause	0	0
Dropped beat	0	0
Ventricular tachycardia	0	0
Salvo	0	0
Triplet	0	0
Couplet	0	0
Bradycardia (=50 bpm)	71	227
Supraventricular tachycardia	0	0
Bigeminy	57	157
Trigeminy	4	7
"R on T"	0	0
Premature aberrant	3246	4698
Isolated aberrant	18	3
Premature normal	11	17
Maximum heart rate	134 (126 – 143)	128 (120 – 137)
Minimum heart rate	51 (48 – 54)	49 (46 – 53)

Data shown are total number of events recorded in each condition over all subjects.

P > 0.05 for all (Wilcoxon matched pairs test). Maximum and minimum heart rates shown as mean (95% confidence intervals), P > 0.05 for both, Student's paired t-test.

During the 2-hour walk, there was no difference in exercise intensity in the presence or absence of the facemask (Table 4) although subjects had a lower systolic blood pressure (114 ± 10 *vs *121 ± 11 mmHg; P < 0.01) when wearing a mask. This was not associated with a change in diastolic blood pressure, heart rate or in heart rate variability measurements.

**Table 4:** Exercise performed and physiological parameters during 2-hour walk.

		Without Mask	With Mask
Activity	Energy expenditure, kcals	340 (314 – 367)	364 (304 – 426)
	Energy expenditure, METS	3.33 (3.09 – 3.57)	3.61 (3.12 – 4.10)
Ambulatory blood pressure	Systolic blood pressure, mmHg	121 (115 – 127)	114* (108 – 120)
	Diastolic blood pressure, mmHg	81 (75 – 87)	79 (74 – 83)
	Mean arterial pressure, mmHg	94 (89 – 99)	90 (86 – 94)
	Heart rate, bpm	88 (82 – 94)	91 (85 – 97)
Heart rate variability	Data validity, %	99.1	97.8
	Average NN interval, ms	594 (562 – 627)	613 (571 – 655)
	pNN50, %	3.3 (0.8 – 5.7)	2.1 (-0.1 – 4.4)
	RMSSD, ms	17.2 (13.4 – 21.0)	20.0 (15.5 – 24.6)
	SDNN, ms	45.8 (36.8 – 54.8)	54.8 (42.5 – 67.0)
	Triangular Index	10.7 (9.1 – 12.4)	11.4 (9.4 – 13.3)
	LF-power, ms^2^	313 (170 – 455)	414 (233 – 595)
	HF-power, ms^2^	76.5 (33.6 – 120.0)	116.8 (52.6 – 181.0)
	LFn, ms	68.2 (60.9 – 75.5)	67.9 (61.9 – 73.9)
	HFn, ms	16.1 (11.9 – 20.3)	16.0 (12.5 – 19.4)
	HF/LF ratio	0.259 (0.173 – 0.344)	0.247 (0.180 – 0.314)

All data expressed as mean (95% confidence interval).

*P < 0.01 compared to control (without mask) day, paired Student's t-test.

P > 0.05 for all other parameters compared to control (without mask) day.

Subjects reported only very minor symptoms (Table 5) during the study period. The mask was generally well tolerated with an average score of 24.8% (95% CI, 16.2 – 33.3%); 0% being completely tolerable and 100% being intolerable.

**Table 5:** Symptom questionnaire.

Symptoms assessed by visual analogue scale (0 – 100)							
	Without mask			With mask			P
	Before walk	After walk	24 hours after walk	Before walk	After walk	24 hours after walk	
	
Headache	4.00 ± 14.13	2.53 ± 5.55	1.13 ± 2.26	3.47 ± 12.06	0.73 ± 1.03	1.13 ± 2.10	n/s
Dizziness	0.40 ± 0.91	1.07 ± 2.22	0.67 ± 1.35	0.47 ± 0.99	0.80 ± 1.57	0.47 ± 0.92	n/s
Tiredness	1.40 ± 4.12	8.47 ± 12.14	9.60 ± 14.78	2.07 ± 5.35	7.40 ± 9.37	2.13 ± 4.10	n/s
Sickness	0.40 ± 0.91	1.07 ± 2.22	0.67 ± 1.35	0.53 ± 0.99	0.87 ± 1.51	0.80 ± 1.32	n/s
Cough	1.07 ± 3.08	1.80 ± 4.80	0.80 ± 1.61	0.73 ± 1.49	1.00 ± 1.73	0.60 ± 1.18	n/s
Difficulty in breathing	0.40 ± 0.91	0.67 ± 0.90	1.13 ± 2.83	3.87 ± 9.23	3.80 ± 8.10	1.60 ± 3.70	<0.05
Irritation of the eyes	1.00 ± 3.09	1.40 ± 3.60	1.13 ± 2.83	1.00 ± 2.59	1.67 ± 3.27	0.87 ± 1.69	n/s
Irritation of the throat	1.00 ± 2.83	1.47 ± 4.07	1.73 ± 4.56	0.73 ± 1.87	1.07 ± 2.63	1.40 ± 2.77	n/s
Irritation of the nose	1.00 ± 2.56	1.53 ± 3.78	1.27 ± 3.58	0.67 ± 1.23	1.07 ± 1.91	0.67 ± 1.35	n/s
Unpleasant smell	0.40 ± 0.74	0.93 ± 1.22	1.00 ± 1.69	0.67 ± 1.23	0.60 ± 0.91	0.80 ± 1.15	n/s
Bad taste	0.53 ± 1.13	0.73 ± 0.96	0.60 ± 0.83	0.40 ± 0.74	0.60 ± 1.18	0.93 ± 1.49	n/s
Difficulty walking		12.53 ± 13.24			15.13 ± 11.51		n/s
Perception of pollution		19.80 ± 18.37			11.60 ± 10.44		n/s

All data expressed as mean ± standard deviation. "Difficulty walking" and "Perception of pollution" tested using paired Student's t-tests. All other variables tested using 2-way ANOVA with repeated measures including time and mask intervention as variables. P value shown is the effect of the mask intervention.

## Discussion

In this study, we have shown for the first time that a simple well-tolerated personal intervention to reduce exposure to airborne particulate air pollution leads to a reduction in systolic blood pressure during exercise and an increase in heart rate variability. If translated into a susceptible population, our findings would suggest that wearing a simple facemask has the potential to reduce the incidence of acute cardiovascular events in cities with high levels of air pollution, and could influence the advice given to patients with chronic cardiovascular diseases.

We tested a range of facemasks that differed widely in their efficiency as particle filters. In general, those masks designed to reduce occupational exposure to dusts were more efficient than those marketed as personal protection to cyclists and pedestrians in an environmental setting. The choice of the mask used in this study was influenced by efficiency and comfort. The chosen mask was very well tolerated by subjects as demonstrated by the visual analogue score, and was predicted to reduce the exposure to particulate matter dramatically. When wearing the masks, the subjects did report slightly greater difficulty breathing whilst walking although this did not reduce the level of exercise undertaken by the subjects. This increased resistance to respiration is unlikely to affect the main study findings since such stresses would be predicted to increase blood pressure rather than reduce it.

Recent studies have confirmed a link between blood pressure and exposure to air pollution. Population-based studies have shown increases in both systolic blood pressure and pulse pressure [17,18] with increasing levels of ambient pollution exposure. Controlled exposure studies to concentrated ambient particles and ozone have demonstrated an increase in diastolic blood pressure during a two-hour exposure [12]. Although there were no differences in blood pressure over the whole 24-hour period, we observed a marked difference in systolic blood pressure with exercise. In both groups, blood pressure increased during exercise compared to the 24-hour average, although this increase was less when wearing a facemask. This, in combination with previous controlled exposure studies [12], suggests that particulate air pollution may augment exercise-induced increases in blood pressure, and that the use of a simple facemask can abrogate this. Exercise induced increases in systolic blood pressure have been linked to myocardial infarction [19] as well as stroke [20], and increased blood pressure is an established major risk factor for the development of both atherosclerosis and cardiovascular mortality [21,22]. The reduction in systolic blood pressure seen in this study is similar to that seen with many antihypertensive agents, which have been shown to reduce major cardiovascular events. Therefore we predict that the use of a facemask in a susceptible population has the potential to reduce the incidence of acute cardiovascular events as well as myocardial ischaemia [13,23].

Heart rate variability is a measure of the variation in the R-R interval on the electrocardiogram. A balance of the parasympathetic and sympathetic nervous systems controls the heart rate in order to maintain a constant cardiac output at rest or to respond to increased demands during exercise. A reduction in heart rate variability occurs in various pathophysiological conditions including hypertension [24], heart failure [25] and diabetes mellitus [26], and predicts cardiovascular outcomes [27]. Previous studies have demonstrated a reduction in measures of heart rate variability, particularly the robust and simple time-domain measurement SDNN following exposure to air pollution [14,15,28-33]. In our study we report an increase in overall heart rate variability (SDNN) when subjects wore a mask, suggesting that wearing a mask can, at least in part, prevent the adverse effects of air pollution exposure on heart rate variability.

LF-power also increased with the use of a mask to prevent exposure to air pollution although interpreting this change is more challenging. LF-power is associated with changes in sympathetic tone, and an increase might suggest an increased contribution of the sympathetic nervous system to basal heart rate control. However, simply wearing the facemask may have had a small effect on the measures of heart rate variability described. As previously discussed, subjects did report an increased resistance to breathing when wearing the facemask that may have increased subject anxiety. This in turn could have increased sympathetic nervous system tone and hence lead to a small increase in LF-power. This is a limitation of our study, and the use of a sham facemask in a blinded fashion would have helped minimise the effect of anxiety on these sensitive outcome measures.

Our study has a number of important public health messages. First we have demonstrated that exposure to ambient air pollution has direct and measurable effects on cardiovascular physiological parameters, even young healthy individuals habitually exposed to such elevated levels. Second we have shown that wearing a facemask can abrogate some of these effects in a short period of time. Particle traps are increasingly being fitted to new vehicles to reduce the emissions of particulate matter, both by mass and number concentrations, and this may well go some way to offsetting the associated health effects. Currently, patients with chronic respiratory and cardiovascular conditions are advised to limit their exposure outdoors on days when ambient air pollution levels are high [34-36]. We have shown that wearing a simple inexpensive and well-tolerated facemask can provide an alternative that may lead to reduced cardiovascular morbidity and mortality. We believe that this intervention now needs to be tested in patients with pre-existing coronary heart disease to define its potential role in reducing the burden of cardiovascular disease in polluted environmental settings.

Our study has a number of important limitations. We recruited young healthy volunteers rather than those most susceptible to the effects of air pollution exposure, such as those with coronary heart disease. Whilst it is likely that our findings will be transferrable to this population, further studies are required to confirm our findings. In addition, it was not possible to assess accurately the efficacy of the mask filter when worn by the subjects. Leaks around the facemask will lead to a reduction in the efficacy of particle filtration [37,38] and therefore our predicted exposures during application of the facemask are likely to be an underestimate. However, despite this, we were still able to demonstrate beneficial cardiovascular effects during their use.

## Conclusion

Air pollution exposure is associated with increased cardiovascular morbidity and mortality, and adverse effects on the cardiovascular system. We have shown for the first time that a wearing a facemask appears to abrogate the adverse effects of air pollution on blood pressure and heart rate variability. This simple intervention has the potential to protect susceptible individuals and prevent cardiovascular events in cities with high concentrations of ambient air pollution.

## Competing interests

The authors declare that they have no competing interests.

## Authors' contributions

JL, NM, KD and DN conceived and participated in the design of the study. JC, JL and LJ carried out the clinical studies and collected the data. JL and NM performed the data analysis and statistical analysis. PF, DL, RA and FC performed the assessment of mask filter efficacy. JL drafted the manuscript, and all authors read and approved the final manuscript.
